# Beyond poverty alleviation: The impact of child support grants on healthcare access and contraception use in South Africa

**DOI:** 10.1016/j.dialog.2025.100228

**Published:** 2025-07-05

**Authors:** Norman Tafirenyika Nhede, Adrino Mazenda, Dymon Gondwe

**Affiliations:** School of Public Management and Administration, University of Pretoria, Private Bag X20, Hatfield, Pretoria 0028, South Africa

**Keywords:** Child support grants, CSG, Contraception use, Family planning, Healthcare access, Social assistance

## Abstract

This study examines the impact of Child Support Grants (CSGs) on access to medical care and contraception use in South Africa, investigating whether social assistance can enhance healthcare access beyond its primary aim of alleviating poverty. While previous research highlights CSGs' poverty reduction and welfare enhancement effects, little is known about their effects on healthcare and reproductive healthcare access, especially given South Africa's healthcare disparities. Using data from the first wave of the 2020 National Income Dynamics Study—Coronavirus Rapid Mobile Survey (NIDS-CRAM), this study employs mediation analysis to analyse the effects of CSG receipt on healthcare and contraception access while controlling for socio-economic factors. The findings indicate a complex relationship. CSGs have a positive but insignificant indirect effect on healthcare and contraception access and a significant negative direct effect, suggesting that the current grant structure may not adequately address existing barriers. The results highlight the need for policy changes, indicating that while CSGs are vital as a social safety net, their effectiveness in improving healthcare access could be enhanced through increased grant amounts and targeted interventions to address healthcare costs and structural barriers.

## 1 Introduction

Social assistance programmes have become a critical element of welfare policy in many developing countries, particularly in sub-Saharan Africa, where poverty, inequality, and poor health outcomes are prevalent [1]. In South Africa, the Child Support Grant (CSG) is one of the most significant social assistance programmes developed to counter poverty effects among low-income households at an annual cost of 1.3 % of the GDP [2]. Since its inception in 1998, the programme has played a critical role in supporting vulnerable children by providing a monthly cash transfer to caregivers. Introduced as a social grant to children under the age of seven, with remarkable controversy following the phasing out of the State Maintenance Grant (SMG) ([3]), the program was launched to assist impoverished young children with the overall objective of rooting out inequality. Due to the success of its implementation, programme coverage was increased to include poor children of up to eighteen years [4], with over 12 million child beneficiaries by 2021 [5].

The Child Support Grant (CSG) has played a crucial role in improving child health metrics in South Africa. While it has positively influenced child nutrition and health, barriers such as a lack of documentation and administrative challenges continue to prevent many eligible families from accessing the grant [5].

Although numerous studies have examined the grant's effects on reducing poverty and improving various socioeconomic outcomes [6,7,8], its potential impact on access to medical care and the use of contraception remains insufficiently explored. Given that several other social assistance programs have been implemented to enhance the lives of underprivileged South Africans alongside the CSG, determining its specific impacts poses a challenge [9].

*This study examines the impact of CSG on these critical aspects of healthcare, contributing to a more comprehensive understanding of the CSG's influence on the well-being of South African children and their caregivers.*

The existing body of literature on the CSG in South Africa has predominantly focused on its role in alleviating poverty and enhancing child welfare. Studies highlight how the CSG has contributed to reducing poverty among households with children by providing a reliable source of income [10,11]. Naidoo et al. [12] analysed the socioeconomic determinants of male contraceptive use in South Africa, indicating that financial stability, potentially influenced by programs like the CSG, can affect reproductive choices. Additionally, Kriel et al. [13] highlighted individual-level factors that influence contraceptive uptake, suggesting that economic support mechanisms may play a role in decisions regarding reproductive health.

In another argument, Udjo [14] and Luthuli et al. [10] found that teenagers who receive the CSG are significantly less likely to become pregnant again compared to those who do not receive the grant. Consequently, Chakraborty et al. [15] suggest that prolonged exposure to the CSG correlates with improved cognitive outcomes later in life, highlighting the grant's potential long-term benefits for caregivers. Still debatable due to operational challenges, the implementation of the National Health Insurance (NHI) Act of 2023 to provide universal healthcare access was necessary to complement the CSG by enhancing healthcare accessibility for low-income households [16].

This study is significant in that it extends the current understanding of the CSG by examining its impact on critical healthcare outcomes for both children and their caregivers. While the CSG has been widely studied in relation to poverty alleviation and educational attainment, its influence on healthcare access, utilisation, and well-being remains underexplored. By focusing on these dimensions, the study addresses a crucial gap in the literature and provides policy-relevant insights into how income support mechanisms can contribute to broader developmental outcomes. The originality of the study lies in its dual emphasis on both children and caregivers, recognising the interdependent nature of health within households, and in its comprehensive approach to evaluating both direct and indirect effects of the CSG on healthcare. This contributes to a more holistic and nuanced understanding of the grant's role in promoting social welfare and reducing health-related vulnerabilities in low-income South African households.

## 2 Literature review

The theoretical basis underpinning the study draws on the Capability Approach lens, as developed by Amartya Sen. It suggests that poverty should not merely be looked at in terms of income deprivation but as a lack of capabilities that enable individuals to lead lives they value [17]. In this view, social assistance programmes, like the CSG, have the potential to enhance capabilities by providing recipients with the resources needed to access essential services, including healthcare and family planning.

Applying this framework, the CSG could increase healthcare access and contraception uptake by improving caregivers' economic capacity. Increased financial resources may reduce the opportunity costs of seeking medical care, including transportation and medication expenses. Furthermore, additional income from grants may lead to more consistent and informed choices regarding family planning as financial barriers are diminished, leading to lower rates of second pregnancy [18]. Hence, the Capability Approach explores how social assistance programmes enhance recipients' well-being beyond mere economic terms, incorporating health and other life-enhancing factors like contraception.

The Health Belief Model (HBM) is another pertinent theory that guides the study. It argues that one's engagement in health-related behaviour, such as seeking medical care or using contraception, is determined by their perceived proneness to health challenges, perceived benefits of taking action, and perceived barriers [19]. By providing a guaranteed income, the CSG could alleviate perceived financial obstacles and act as a cue to action, encouraging caregivers to seek health services for themselves and their children. Therefore, the interaction between the Capability Approach and the HBM offers a comprehensive understanding of how economic interventions, like CSGs, can affect healthcare-seeking behaviours and family planning decisions.

**Fig. 1:**
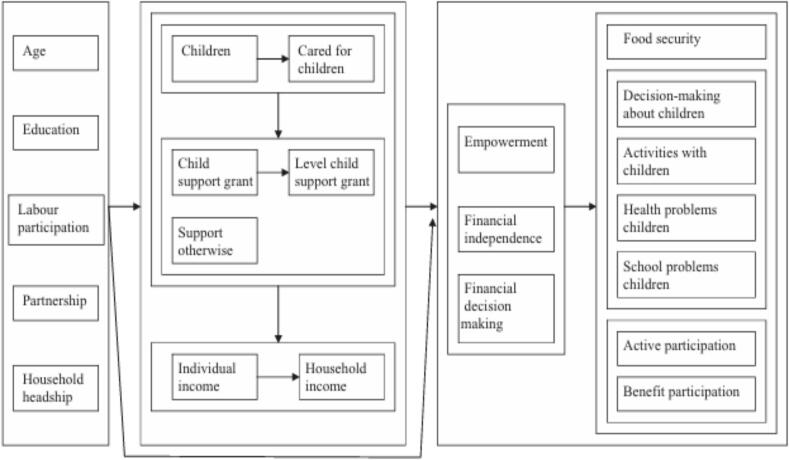
Conceptual framework of child support grants' effects on various socio-economic outcomes.

Fig. 1 shows the conceptual framework of the relationship between the child support grants and various socio-economic outcomes like financial, health, education, nutrition and others.

(Source: Patel et al. [4])

As shown in Fig. 1, receipt of CSG should theoretically cause improvements in household income and lead to improvements in food availability, health and schooling outcomes as well as overall improvements in decision-making about children. The mechanism is that grants can directly alleviate financial constraints by supplementing household income, allowing families to meet basic needs. With improved household income, food security tends to improve, as families can afford a stable and nutritious diet. This is particularly important for children whose physical and cognitive development relies on consistent and adequate nutrition. Consequently, better nutrition can lead to improved health outcomes, reducing the incidence of malnutrition-related illnesses and enhancing overall family growth.

Empirical literature across low– and middle-income countries reveals a strong positive impact of cash transfers on child health and nutrition. The effectiveness was notably higher in programs with conditionalities, such as mandatory attendance at healthcare facilities. The success of these programs was contingent upon robust supply-side mechanisms, ensuring functional and quality services at health centres and schools [20].

Shah et al. [21] found significant heterogeneity in maternal healthcare utilisation in Indonesia, with greater benefits observed in areas with better healthcare infrastructure and among households with varying poverty levels. This underscores the importance of contextual factors in the implementation and outcomes of cash transfer programs.

[22] found that various social protection programmes implemented in Sub-Saharan Africa contribute to inclusive growth. This growth translates to a reduction in inequalities through increased opportunities for the economically marginalised sections of the population to access healthcare, education, improved dietary uptake and enhanced social integration. In another argument from Namibia, Valombola and Omomowo [23] found that while the grants provided essential financial support, they were insufficient to cover healthcare expenses, indicating a need for complementary interventions to enhance healthcare access and outcomes.

In the South African context, Ohrnberger et al. [24] CSGs were associated with improved mental health, significantly reducing depressive symptoms among the poor populations. Similarly, results by Chakraborty et al. [15] indicate that CSGs are also associated with other substantial effects by conferring higher cognitive abilities of recipient mothers.

These studies suggest that grants are essential in enhancing healthcare access, although their focus has been more on general health outcomes rather than specific aspects like contraception use.

Concerning contraception and pregnancy, much as it is known that receipt of a child support grant hardly incentivises pregnancy among teenage mothers [18,14], there is limited but emerging research exploring the relationship between income transfers and pregnancy. Moolla and Goldstein [25] and Victora et al. [26] reported that undernutrition during pregnancy results in stunting, negatively impacting social and health later in life. By providing additional resources, social grants could provide a sustainable mechanism for mitigating these challenges, leading to better health outcomes. However, there is still a scarcity of empirical studies specifically linking CSG receipt with changes in healthcare access and contraception use. The limited focus on this dimension means that much remains to be understood regarding how economic empowerment through social grants influences reproductive health choices.

## 3 Materials and methods

### 3.1 Data and sampling

The data was derived from the nationally representative first wave of the National Income Dynamics Study—Coronavirus Rapid Mobile Survey (NIDS-CRAM) collected in 2020. This is a telephone follow-up survey of a subset of individuals from NIDS Wave 5 of 2017. This survey centres on individuals in South Africa and provides a representative sample of the entire nation. Participants are contacted and asked about various topics, including their income, employment status, household well-being, and receipt of grants. The Southern Africa Labour and Development Research Unit (SALDRU) collected the data utilised in this article.

The information was gathered as part of initiatives to examine the effects of the physical limitations linked to the State of Disaster declared in South Africa in March 2020, along with the socioeconomic results of the COVID-19 pandemic. NIDS-CRAM was initially created as a component of a larger research project known as the Coronavirus Rapid Mobile Survey (CRAM), which aimed to guide policy with trustworthy information on income, employment, and welfare. The sample was created through a stratified sampling method and includes individuals who were aged 18 or above during the preparation for the first wave of NIDS-CRAM fieldwork in 2020.

#### 3.1.1 Variables

Access to medical services and contraception is the study's dependent variable, which is of a dummy nature. This variable was aimed at determining whether an individual had had challenges accessing medication and contraception during the previous four weeks. Information regarding whether somebody received a CSG or not during the same period constituted the independent variable. From this information, a binary variable was generated with 1 representing CSG access and 0 otherwise. Out of the five National Income Dynamics Study, Coronavirus Rapid Mobile Survey (NIDS-CRAM) waves, the dependent variable is available in the first wave only, which explains the use of that wave only in this study. The household disposable income, province of residence, household size and composition, education level, number of grants received, COVID-19-induced income loss and COVID-19-induced food insecurity dummies constitute the study's other independent variables.

Household disposable income is a crucial determinant of socioeconomic status [27]. This includes healthcare access and contraception use, as it directly influences a household's ability to afford medical services, transport to clinics, and contraceptive products. Other factors constant, lower-income households may face financial barriers, while higher disposable income households could facilitate better healthcare utilisation. The province of residence accounts for regional disparities in healthcare infrastructure, service availability, and cultural attitudes towards contraception. South Africa's provinces vary significantly in development and resource allocation, making geographic location a key factor in understanding access disparities. Household size and composition affect resource distribution within the household [28], influencing whether funds are prioritised for healthcare or other needs. Larger households may spread resources thinly, while the presence of more children or elderly members could shift spending priorities away from contraception.

Education level is strongly linked to health literacy [29] and awareness of contraceptive options. Higher education often correlates with better knowledge of sexual health services and greater autonomy in healthcare decision-making, particularly for women. On the other hand, the number of grants received reflects the extent to which a household relies on social assistance, which may buffer against financial constraints. More grants could improve healthcare access by supplementing income, but overlapping grants may also indicate deeper socio-economic vulnerabilities. COVID-19-induced income loss captures the pandemic's economic shock, which may have disrupted healthcare access due to job losses or reduced earnings. This variable helps assess whether financial strain during the pandemic exacerbated existing barriers to contraception and medical care. COVID-19-induced food insecurity is included because households prioritising food over healthcare may neglect contraception or medical visits. The pandemic worsened food insecurity for many, potentially diverting limited resources away from health-related expenditures.

Each variable thus provides a distinct lens through which to analyse how child support grants interact with broader socio-economic factors to influence healthcare access and contraceptive behaviour.

### 3.2 Mediation analysis

This study is supported by data from the first wave of the National Income Dynamics Study—Coronavirus Rapid Mobile Survey (NIDS-CRAM) collected in 2020 and has a nationally representative sample of 3634 respondents. Mediation analysis was adopted as it allows for the decomposition of the total effect into direct and indirect pathways. This approach is particularly relevant in this context because the receipt of child support grants (independent variable, X) may influence access to medical care and contraception (dependent variable, Y) both directly and indirectly through the number of grants received (mediating variable, M). According to Imai et al. [30], mediation analysis provides such a framework to quantify these pathways since it offers crucial insights into the mechanisms through which interventions impact outcomes. While numerous policy evaluation mechanisms have been developed, mediation analysis is also crucial as it allows for the disentangling of effects into indirect effects through mediators [31].

The suitability of mediation analysis is further supported by its ability to address endogeneity and confounding, which are common challenges in evaluating social programmes. A study by Ricotta et al. [32] highlights the use of mediation analysis in similar contexts, demonstrating its robustness in identifying causal pathways in health-related outcomes. This study adopts a two-regression econometric methodology highlighted by Jung [33] as follows:

$$Y=\beta_{0}+B\left(E\right)+e$$

Where a simple regression analysis is run with E on Y in order to estimate path c’. Secondly, a multivariate regression is carried out with E and M in order to predict the outcome (Y) as follows:

$$Y=\beta_{0}+\beta_{1}\left(E\right)+\beta_{2}\left(M\right)+e$$

Where the coefficient *β* represents the total effects, the direct effect from E to Y c’ corresponds to $\beta_{1}$ and the indirect effect ($\beta_{\mathrm{indirect}}=\beta -\beta_{1}).$

Robust standard errors generated through bootstrapping are utilised to improve the estimation, and thus reduce bias [34]. The mediation analysis was undertaken using the GLM mediation package in the Jamovi software.

In order to check for the presence of multicollinearity caused by a strong linear relationship between at least two independent variables [35], a variance inflation factor (VIF) test was undertaken by regressing the dependent variable on the predictors, followed by the VIF test as proposed by Kyriazos and Poga [36]. The VIF values for all the variables used were less than 10, indicating the absence of multicollinearity. The test results are presented in Table 1 below.

**Table 1:** Multicollinearity test using the variance inflation factor.

Variable	VIF	1/VIF
Number of grants received	2.90	0.35
Grant receipt dummy	2.41	0.41
Household size	1.77	0.56
Natural log of household income	1.37	0.73
Population group	1.31	0.76
Food insecurity dummy	1.19	0.84
Income loss dummy	1.12	0.89
Residents older than 60 years	1.11	0.90
Gender	1.09	0.92
Province	1.07	0.93
Highest school grade completed	1.06	0.94
Mean VIF	1.49	

Source: Authors' Stata iterations.

## 4 Results

### 4.1 Descriptive statistics

Table 2 shows the respondents' distribution across the variables being studied.

**Table 2:** Descriptive statistics.

	N	Mean	Std. Dev.	Min	Max
Gender (1 = Male)	3634	0.34	0.47	0	1
Natural log of household disposable income	3634	8.02	1.08	0.69	12.43
Province	3634	5.06	2.40	1	9
Residents older than 60 years	3634	0.48	0.71	0	4
Household size	3634	4.97	2.62	1	14
Population group (1 = B, 2 = C, 3 = A/I, 4 = W)	3634	1.29	0.74	1	4
Highest school grade completed	3634	10.34	3.17	0	19
Number of grants received	3634	1.41	1.47	0	8
Grant receipt dummy (1 = Yes)	3634	0.63	0.48	0	1
COVID-19 income loss dummy (1 = Yes)	3634	0.42	0.49	0	1
COVID-19 food insecurity dummy (1 = Yes)	3634	0.53	0.50	0	1
Medication and contraception dummy (1 = Yes)	3634	0.27	0.44	0	1

Source: Authors' Stata iterations. On population group, 1, 2, 3 and 4 represent Black, Coloured, Asian/Indian and White, respectively.

As shown in Table 2, the highest reported household income was R25,000, with a mean of R5,905 and a standard deviation of R11,200. Considering that income data is usually right-skewed since there are few high-income individuals compared to the rest, the income data in the table above has been presented in natural logarithmic form. Logging is also essential as it simplifies results interpretation since it enables explanation of the same in terms of approximate percentage changes.

Most respondents (approximately 63 %) reported receiving a child support grant from the government, while 42 % and 53 % reported having lost income and, therefore, experiencing food insecurity, respectively. At least 43 % of the respondents had achieved the grade 12 level of schooling. Although it is possible to receive multiple CSGs, the average number received was 1.4.

The mean household size was approximately five individuals per household in as much as those with as many as fourteen individuals were recorded. Access to medication was the lowest at a rate of 27 %. The Black community constituted the majority of the respondents, while the highest number of the elderly associated with a particular household was four.

### 4.2 Empirical findings

The results present the indirect, direct, and total effects. Indirect effects show the relationship component between an independent and dependent variable transmitted through an intervening construct [37]. This path shows how the independent variable influences the dependent one through the mediator. Direct effects report relationships between the independent variable and the dependent one. However, it is worth noting that both the direct and indirect effects do not necessarily represent causal quantities (in the same sense as average treatment effects, even in the event of random assignment of subjects to treatment conditions [38]. For this study, the number of grants received was used as a mediator. Theoretically, receiving multiple grants may signify increased household financial capacity, allowing beneficiaries to meet healthcare needs better and access reproductive services [7].

Table 3 shows the mediation analysis findings with the total grants received as the mediator.

**Table 3:** Mediation results: Mediator- Total number of grants received.

			95 % confidence interval		Significance	
Effect type	Variable	Estimate	Lower	Upper	*p*-value	
Indirect	Grant receipt	0.01376	−0.01619	0.04366	0.343	
	Gender	−0.00054	−0.00202	0.00071	0.386	
	Natural log of household income	0.00036	−0.00045	0.00144	0.367	
	Province of residence	0.000032	−0.00001	0.00024	0.594	
	Residents older than 60 years	−0.00092	−0.00304	0.00101	0.35	
	Household size	0.00171	−0.00208	0.00553	0.343	
	Population group	−0.00013	−0.00072	0.00022	0.558	
	Highest grade completed	−0.00004	−0.00026	0.0001	0.488	
	COVID-19 income loss dummy	−0.00028	−0.00157	0.00051	0.464	
	COVID-19 food insecurity dummy	0.00026	−0.00057	0.00148	0.488	
Direct	Grant receipt	−0.04232	−0.08578	0.00747	0.071	**
	Gender	−0.06835	−0.10133	−0.0379	<0.001	***
	Natural log of household income	−0.00158	−0.01735	0.01365	0.842	
	Province of residence	0.00548	−0.00282	0.01103	0.082	**
	Residents older than 60 years	0.0157	−0.0064	0.03644	0.148	*
	Household size	0.00872	0.00065	0.01666	0.019	**
	Population group	−0.05083	−0.06989	−0.02844	<0.001	***
	Highest grade completed	−0.00383	−0.00903	0.00118	0.107	*
	COVID-19 income loss dummy	0.03118	−0.00066	0.06073	0.047	**
	COVID-19 food insecurity dummy	−0.00959	−0.04119	0.02183	0.548	
Total	Grant receipt	−0.02855	−0.06605	0.00705	0.121	*
	Gender	−0.06889	−0.09768	−0.03964	<0.001	***
	Natural log of household income	−0.00122	−0.01767	0.01417	0.877	
	Province of residence	0.00551	−0.00017	0.01141	0.08	**
	Residents older than 60 years	0.01478	−0.00678	0.03673	0.172	*
	Household size	0.01043	0.00329	0.01708	0.001	***
	Population group	−0.05097	−0.07081	−0.0321	<0.001	***
	Highest grade completed	−0.00387	−0.00878	0.00101	0.103	*
	COVID-19 income loss dummy	0.03089	−0.00089	0.06503	0.049	**
	COVID-19 food insecurity dummy	−0.00934	−0.04293	0.02109	0.558	

Confidence intervals computed with Bootstrap percentiles. Asterisks ***, ** and * denote 1 %, 5 % and 10 % significance level, respectively.

#### 4.2.1 Grant receipt

The indirect effect of receipt of a grant is positive but statistically insignificant (estimate = 0.01376, p = 0.34). However, the direct effect of a grant receipt is both negative and statistically significant (estimate = −0.04232, *p* = 0.071), suggesting the existence of some inverse association between grant receipt and access to medication and contraception. The total effect is also negative and statistically significant, confirming the existence of the inverse relationship between the two variables (estimate = −0.02855, *p* = 0.121).

#### 4.2.2 Gender

The indirect effect between gender and child support grants is both negative and statistically insignificant (estimate = −0.0005, *p*-value = 0.386). On the other hand, the direct and total effects are both negative but statistically significant (estimate = −0.04232 and *p* = 0.071) and (estimate = −0.029, *p* = 0.121), respectively. This suggests that receipt of grants is associated with a decrease in medical care and contraception access.

#### 4.2.3 Household income

The indirect effect between household income and child support grants is positive, but statistically insignificant (estimate = 0.00036, *p* = 0.367). The same insignificant relationship is observed between the two variables on the direct and total effects (estimate = −0.00158, *p* = 0.842) and estimate = −0.00122, *p* = 0.877, respectively.

#### 4.2.4 Province of residence

An insignificant relationship is observed on the indirect effects between the province of one's residence and the CSG's effect on medical and contraception access (estimate = 0.000032, *p* = 0.594). However, the relationship on both direct and total effects is both positive and significant (estimate = 0.00548, *p* = 0.082) and (estimate = 0.00551, p = 0.08), suggesting that one's province of residence is directly associated with access to medical care and contraception because of exposure to CSGs.

#### 4.2.5 Number of residents aged over 60

The indirect effects indicate an insignificant and negative relationship between CSG receipt and the number of residents over 60 years in a household and medical and contraception access (estimate = −0.00092, *p* = 0.35. However, the direct effects indicate a positive and significant relationship (estimate = 0.0157, *p* = 0.148), suggesting that as one's age is positively associated with increased need for expenditure on medical and contraception.

#### 4.2.6 Household size

There is an insignificant and positive indirect effect between household size and medical and contraception access (estimate = 0.00171, *p* = 0.343). However, the direct effect indicates a significant relationship between household size and medical and contraception access (estimate = 0.00872, *p* = 0.019), suggesting that the size of the household is associated with medical and contraception decisions as a result of receipt of CSGs.

#### 4.2.7 Population group

There is an insignificant indirect effect between population group and medical and contraception access (estimate = −0.00013, *p* = 0.558). On the other hand, the direct effects indicate a negative but significant relationship between population group and medical and contraception access (estimate = −0.05083, *p* ≤0.001), suggesting that population group is associated with decisions on medical and contraception access as a result of exposure to CSGs.

#### 4.2.8 Schooling effect

The indirect effects indicate a negative and insignificant relationship between schooling accomplishment and medical and contraception access (estimate = −0.00004, *p* = 0.488). The direct effects path between the highest schooling grade indicates a negative but significant relationship (estimate = −0.00383, *p* = 0.107), suggesting that schooling accomplishment is associated with a reduction in medical and contraception access decisions as a result of accessing CSGs.

#### 4.2.9 COVID-19 income loss dummy

The indirect effects indicate a negative but insignificant relationship between income lost (estimate = −0.00028, *p* = 0.464) and medical and contraception access. The direct and total effects indicate a positive and significant relationship between the two (estimate = 0.03118, *p* = 0.047) and (estimate = 0.03089, *p* = 0.049), respectively, indicating that income loss has a bearing on medical and contraception access decisions.

#### 4.2.10 COVID-19 food insecurity dummy

On the indirect effects path, there is a positive but insignificant relationship between COVID-19-induced food insecurity (estimate = 0.00026, p = 0.488) and medical and contraception access. The direct and total effects path between the two is also insignificant (estimate = −0.00959, *p* = 0.548) and (estimate = −0.00934, *p* = 0.558), respectively.

## 5 Discussion

This study employs the mediation analysis to examine the direct and indirect effects of CSG receipt on healthcare and contraceptive access, exploring how variables such as gender, province, household composition, population group, education, and pandemic-induced income loss mediate these relationships. The findings reveal complex, and in some cases contradictory, dynamics that call into question the capacity of CSGs to deliver meaningful improvements in healthcare equity.

Social protection systems in South Africa, particularly the CSG, have long been positioned as critical tools in addressing poverty and improving welfare outcomes for vulnerable populations. While the CSG has contributed to alleviating financial hardship in many households, its broader implications for access to healthcare and contraception remain contested.

The core findings of this study point to a statistically significant negative direct effect of CSG receipt on access to medical care and contraception. This suggests that, independent of intermediary factors, receiving the grant is associated with diminished access to health services. Conversely, the indirect effect, although positive, lacks statistical significance, indicating that while CSGs may initiate supportive processes through mediating variables, these processes are not strong enough to drive meaningful change [39,40]. This paradox underscores the reality that while CSGs contribute to household income, their limited value relative to the cost of living may explain the insufficient improvement in healthcare access. As Hajdu et al. [7] note, the grant was never intended to transform household economic status but rather to safeguard child welfare, a focus that limits its broader developmental utility.

Gender, often a salient variable in studies of health access, was found to have a negative and statistically insignificant moderating effect in this analysis. Although gender-based disparities in healthcare utilisation are well documented, this finding suggests that the impact of CSGs on health access does not significantly differ by gender. It may reflect the more dominant influence of socio-economic constraints, such as unemployment, distance from health facilities, and structural inequalities, on healthcare access, which overshadow gendered differences in service utilisation [41,40].

The study also identifies the province of residence as a significant direct predictor of healthcare and contraception access. Despite the lack of a significant indirect effect through CSG receipt, the positive direct and total effects highlight the role of geographical context in determining the effectiveness of social grants. Provinces with better infrastructure, coordinated service delivery, and responsive health systems may enable grant recipients to leverage these services more effectively [7]. The disparities in provincial service delivery capacities point to the need for region-specific strategies to enhance healthcare access, a finding aligned with recent research on provincial poverty and expenditure effectiveness [42].

In terms of household composition, households with members over 60 years of age were found to have a positive and significant direct effect on access to medical services and contraception. This may reflect increased demand for health services in multigenerational households, where older adults often require regular care. The presence of elderly dependents can reconfigure household priorities and expenditures, including those related to reproductive health [43,44]. Similar patterns emerge in relation to household size, where larger households show a significant direct association with healthcare access. These results are consistent with the work of Kneale et al. [45], who argue that CSGs may help mitigate financial stress in larger families, thereby facilitating access to health services, though the effect is mediated by internal family dynamics and resource distribution [46,47].

Race and population group remain critical lenses for interpreting inequities in healthcare access in South Africa. The analysis finds that while population group has no statistically significant indirect effect via CSG receipt, its direct effect is significantly negative. This finding confirms the persistence of systemic racial disparities in healthcare access, which are rooted in historical structural inequalities and cultural barriers [48,49]. The inability of CSGs to mitigate these disparities suggests that grants alone are insufficient to dismantle entrenched social hierarchies and that broader structural reforms are required to promote healthcare equity.

An unexpected result arises with educational attainment, where higher schooling levels are associated with decreased access to medical and contraceptive services. This counterintuitive outcome may be attributed to opportunity costs, such as time constraints or migration for work and study, which reduce healthcare utilisation despite increased knowledge or awareness [18]. It challenges the assumption that education invariably improves health-seeking behaviour and calls for a nuanced understanding of how socio-economic pressures can interfere with access, even among the more educated.

The analysis also explores the role of economic shocks, specifically income loss during the COVID-19 pandemic. Surprisingly, income loss has a positive and statistically significant direct and total effect on access to healthcare and contraception. This could be due to government relief packages or community-based interventions that temporarily offset income loss and facilitated access to healthcare [50]. However, the insignificant indirect effect through CSG receipt indicates that the grant alone was not a sufficient buffer during the crisis. As Kerschbaumer et al. [42] and Khambule [50] argue, the majority of COVID-19 relief bypassed the informal sector, where most South Africans work, further limiting the effectiveness of the CSG in this context.

Taken together, these findings expose the limitations of the CSG as a tool for improving healthcare and reproductive service access in South Africa. While it remains a vital social protection mechanism, its design and scale are not adequate to address broader socio-economic and structural challenges. Recent policy evaluations have similarly concluded that grants need to be integrated with other developmental interventions, such as transportation subsidies, expanded clinic networks, and public health education campaigns, to yield significant outcomes [51].

## 6 Conclusions

The CSG has long been a cornerstone of South Africa's social protection strategy, aimed at alleviating poverty and improving the well-being of children in low-income households. While studies indicate that CSGs may have a modest, indirect positive effect on healthcare access and contraceptive use, these effects are often statistically insignificant. More concerning is the observation that the direct effect of CSG receipt on healthcare and reproductive service access is negative and statistically significant. This suggests that despite their widespread reach, CSGs are not effectively improving access to essential health services, likely due to insufficient grant amounts and entrenched socio-economic barriers. To address these shortcomings, localised interventions are essential to ensure that the grants meaningfully support improved health outcomes across different regions of the country.

One of the most pressing issues highlighted by recent findings is the significant regional disparity in healthcare access among CSG recipients. Provinces such as the Western Cape and Gauteng often demonstrate better access, while others, including the Eastern Cape, Limpopo, and KwaZulu-Natal, continue to lag behind. This uneven impact underscores the need for targeted, province-specific interventions that are sensitive to local conditions and infrastructural realities.

A key strategy would be the introduction of province-specific CSG top-ups in underperforming areas. These top-ups would provide additional financial support specifically aimed at offsetting the unique costs faced by households in these regions, such as transportation to distant clinics or the purchase of out-of-pocket medications. This approach recognises that the standard national CSG amount may not adequately cover the diverse cost burdens associated with healthcare access in different locales.

Another important intervention is the deployment of mobile health clinics and community outreach services in rural and hard-to-reach communities. These services can deliver primary healthcare, maternal health services, and family planning support directly to beneficiaries, reducing the need for travel and minimising associated expenses. Community-based health outreach can foster synergies between social protection and health service delivery, particularly when integrated with CSG payment points or community centres.

In addition to improving physical access to healthcare, health literacy programmes are essential in regions where low levels of education inhibit the effective use of available services. These programmes can be delivered through schools, local non-governmental organisations, and community workshops to educate caregivers, particularly women, on the importance of regular health check-ups, immunisation, and contraceptive options. Better-informed recipients are more likely to seek care proactively, thereby enhancing the effectiveness of CSGs in promoting child and maternal health.

Addressing infrastructure gaps also requires public-private partnerships aimed at expanding the capacity and reach of healthcare services. By incentivising private investment through subsidies or shared use of facilities, provincial governments can boost healthcare provision in areas where public services are insufficient. Such partnerships can also help alleviate pressure on overstretched public clinics, improving service quality for all users.

Furthermore, in provinces where geographic inaccessibility poses a substantial barrier, transportation subsidies should be considered as a complementary support mechanism. Linking these subsidies to CSG eligibility can help ensure that poor households are not excluded from healthcare simply because of distance or high transport costs.

To ensure that these interventions are data-driven and responsive to evolving needs, enhanced provincial data collection and monitoring systems must be established. These systems should capture disaggregated data on healthcare usage, household composition, and socio-economic status to inform targeted adjustments in policy implementation.

Equally important is the need for cultural sensitivity and language accessibility in healthcare service delivery. In provinces with diverse linguistic and cultural profiles, services must be delivered in local languages and by personnel who understand and respect community norms. Training community health workers from within the local population can enhance trust and uptake of services among CSG recipients.

Finally, effective localisation of CSG-related health interventions requires multi-sectoral coordination at the provincial level. Departments of social development, health, education, and transport must work collaboratively to design and implement integrated service delivery models. Such coordination ensures that interventions are not fragmented and that beneficiaries experience a coherent and comprehensive support system.

## 7 Limitations

This study, based on secondary data from the COVID-19 period, faces limitations that affect generalizability and validity. Pandemic-related disruptions may distort the typical relationship between CSGs and health outcomes. Limitations include potential omitted variable bias, recall bias in self-reported data, and exclusion of marginalised groups. Future research should adopt mixed methods to capture contextual factors, use longitudinal designs to assess changes over time, and apply robustness checks to address endogeneity. Triangulating data with administrative records and situating findings within the pandemic context will enhance relevance and accuracy, informing more resilient and inclusive social protection policies.

## CRediT authorship contribution statement

**Norman Tafirenyika Nhede:** Writing – review & editing, Writing – original draft, Conceptualization. **Adrino Mazenda:** Writing – review & editing, Writing – original draft, Methodology, Conceptualization. **Dymon Gondwe:** Writing – review & editing, Writing – original draft, Conceptualization.

## Declaration of competing interest

The authors declare that they have no known competing financial interests or personal relationships that could have appeared to influence the work reported in this paper.
